# A multicenter retrospective study on anesthesia methods and their impact on neurocognitive outcomes and other complications in elderly patients undergoing hemiarthroplasty

**DOI:** 10.3389/fmed.2025.1599989

**Published:** 2025-08-11

**Authors:** Gengrui Zhong, Xiaoli Huang, Congye Li, Deqiang Wang, Dingding Huang, Menghan Sun, Quanhong Zhou, Yong Guo

**Affiliations:** ^1^Department of Critical Care Medicine, Sixth People's Hospital Affiliated to Shanghai Jiao Tong University School of Medicine, Shanghai, China; ^2^Department of Critical Care Medicine, Jinshan Branch of Shanghai Sixth People’s Hospital (Shanghai Jinshan District Central Hospital), Shanghai, China; ^3^Department of Critical Care Medicine, South Branch of Sixth People's Hospital Affiliated to Shanghai Jiao Tong University School of Medicine (Shanghai Fengxian Central Hospital), Shanghai, China; ^4^Department of Anesthesiology, South Branch of Sixth People's Hospital Affiliated to Shanghai Jiao Tong University School of Medicine (Shanghai Fengxian Central Hospital), Shanghai, China; ^5^Department of Critical Care Medicine, Xuhui Branch of Sixth People's Hospital Affiliated to Shanghai Jiao Tong University School of Medicine (Shanghai Eighth People's Hospital), Shanghai, China

**Keywords:** anesthesia methods, elderly patients, hip fracture, postoperative delirium, delayed neurocognitive recovery

## Abstract

**Objective:**

To evaluate the effects of various anesthesia techniques on perioperative neurocognitive disorders (PND) and other postoperative complications in elderly patients undergoing hemiarthroplasty for hip fractures.

**Methods:**

This multicenter retrospective observational study analyzed 5,005 elderly patients (≥65 years) with hip fractures who underwent hemiarthroplasty and had complete perioperative clinical data. Patients were categorized into five anesthesia groups: a, Combined intravenous-inhalation anesthesia (IVA + IHA); b, IVA + IHA with peripheral nerve block (PNB); c, Intravenous anesthesia (IVA) with PNB; d, Spinal anesthesia (SA); e, SA with PNB. Postoperative delirium (POD) was assessed twice daily during the first 3 postoperative days using the Confusion Assessment Method (CAM). Delayed neurocognitive recovery (DNR) was evaluated via telephone follow-up on postoperative day 7. Other postoperative complications, as well as 30-day and 6-month mortality rates, were systematically recorded.

**Results:**

The analysis revealed no significant differences in POD incidence among the first three anesthesia groups (a/b/c) or between the last two groups (d/e) (*p* > 0.05). However, when comparing the combined first three groups with the combined last two groups, the difference was statistically significant (*p* < 0.05), with an overall *p*-value of 0.029. No significant differences were observed in DNR incidence among the five groups (*p* = 0.12), indicating that anesthesia methods significantly affected POD occurrence but not DNR. Significant differences were found in postoperative pulmonary infection (PI) rates among the five anesthesia groups (*p* = 0.0314). The overall PI incidence was significantly higher in general anesthesia groups compared to regional anesthesia groups, with notable differences in pairwise comparisons. However, no significant differences were observed in urinary tract infection (UTI), deep vein thrombosis (DVT), pulmonary thromboembolism (PTE), or mortality rates among the groups (*p* > 0.05). SA & SA + PNB (de) significantly reduced POD risk: SA: OR 0.3239 (95% CI 0.2215–0.4735), 67.61% risk reduction; SA + PNB: OR 0.3634 (95% CI 0.2966–0.4452), 63.66% risk reduction (Both statistically significant, CI excludes 1). IVA + IHA: OR 1.3929 (95% CI 1.0590–1.8320) suggested potential PI risk increase, but wider CI indicates lower certainty.

**Conclusion:**

These findings suggest that regional anesthesia may be associated with lower early POD and pulmonary infection rates. Further prospective randomized controlled trials are needed to validate these results.

## Introduction

Hip fractures in elderly patients represent a significant global public health concern, with escalating incidence attributed to aging populations and rising osteoporosis prevalence (1, 2). These injuries carry substantial morbidity and mortality, with in-hospital mortality rates of 5% and 30-day mortality reaching 10% (3, 4). The 2018 reclassification of perioperative neurocognitive disorders (PND) established four distinct categories: pre-existing cognitive impairment, postoperative delirium (POD) occurring within 7 days, delayed neurocognitive recovery (DNR) within 30 days, and postoperative cognitive dysfunction (POCD) persisting up to 1 year postoperatively (5). POD demonstrates particular clinical significance due to its association with prolonged hospitalization, accelerated cognitive decline, and increased mortality (6–10).

Perioperative complications extend beyond neurocognitive outcomes, encompassing pulmonary infections related to mechanical ventilation and surgical duration, catheter-associated urinary tract infections, and venous thromboembolic events including deep vein thrombosis and pulmonary embolism. These concurrent complications collectively contribute to the elevated mortality observed in this patient population (11, 12).

The optimal anesthetic approach for geriatric hip fracture surgery remains controversial. While historical data from Rodgers et al. suggested potential mortality benefits with neuraxial techniques (13, 14), contemporary studies accounting for recent advancements in peripheral nerve blockade (PNB) and ultrasound guidance remain limited (15, 16). Modern anesthesia practice increasingly utilizes hybrid techniques combining PNB with monitored anesthesia care, though comparative effectiveness data against traditional general or spinal anesthesia are lacking.

This study aims to compare the differential effects of five anesthetic techniques on the incidence of perioperative neurocognitive disorders (PND) as the primary outcome, as well as secondary outcomes (including pulmonary infections, urinary tract infections, pulmonary embolism, deep vein thrombosis, and mortality) in elderly patients undergoing hemiarthroplasty. We hypothesize that regional anesthesia techniques (spinal anesthesia with or without peripheral nerve blocks) will demonstrate superior outcomes in reducing early postoperative delirium and pulmonary infection rates compared to general anesthesia.

## Methods

### Study design

This multicenter retrospective study analyzed data from elderly patients undergoing hemiarthroplasty between January 2021 and December 2024 to evaluate the impact of different anesthesia techniques on perioperative neurocognitive disorders [specifically postoperative delirium [POD] and delayed neurocognitive recovery (DNR)], along with postoperative complications including pulmonary infections, urinary tract infections, deep vein thrombosis, and pulmonary embolism. All data, including assessments for postoperative complications, were extracted from pre-existing Surgical Patient Database at participating hospitals. No prospective interventions or dedicated research-specific assessments were conducted. This retrospective study utilized de-identified data extracted from pre-existing clinical databases. The Ethics Committee of Shanghai Sixth People’s Hospital waived the requirement for informed consent [Approval No. 2021–274-(1)] in accordance with institutional policies and national regulations, as no prospective contact with patients occurred. All patient identifiers (e.g., names, ID numbers) were removed prior to analysis. Data were accessed via a secured, password-protected hospital server under institutional data governance protocols, with analysis restricted to coded study numbers.

### Inclusion criteria

1. Patients aged 65 years or older.
2. Patients with hip fractures undergoing hemiarthroplasty.

### Exclusion criteria

1. Patients with preoperative Mini-Mental State Examination (MMSE) scores less than 24, as the occurrence of perioperative neurocognitive disorders was the primary outcome of our study.
2. Patients with a history of psychiatric illness.
3. Patients taking antidepressants.
4. Surgery duration exceeding 3 h (indicating surgical failure or Selected complex surgical scenarios).
5. Patients diagnosed with multiple fractures or those who underwent a second surgery or infection during hospitalization.
6. Patients with language barriers or those who speak foreign languages.
7. Patients with alcohol or drug dependence.
8. Patients with severe visual or hearing impairments.
9. To eliminate the impact of surgical methods, patients who only received internal fixation, total hip replacement, or no surgery were excluded.
10. Patients lost to follow-up.

### Anesthesia management

Anesthesia techniques followed uniform implementation protocols across all centers

a. Intravenous-inhalation general anesthesia (induction with 0.5 μg/kg sufentanil, 1 mg/kg rocuronium, and 2 mg/kg propofol; maintenance with 1–2% sevoflurane inhalation, and additional 5–10 μg sufentanil before skin incision);
b. Intravenous-inhalation general anesthesia combined with PNB at different sites (using 100 mg ropivacaine);
c. Intravenous anesthesia (induction with 0.5 μg/kg sufentanil, 1 mg/kg rocuronium, and 1 mg/kg propofol; maintenance with additional 5–10 μg sufentanil before skin incision) combined with PNB;
d. Spinal anesthesia (intrathecal injection of 20 mg ropivacaine diluted in cerebrospinal fluid, slowly administered over 3–5 mL);
e. Spinal anesthesia combined with PNB (peripheral nerve block). Each anesthesia method followed its standard protocol to ensure consistency among participants.

The anesthesiologists in our hospital and affiliated medical consortium hospitals select anesthesia techniques based on patients’ conditions. General anesthesia is prioritized as the first-line option due to its safety profile, while patients with respiratory insufficiency receive peripheral nerve block (PNB) to avoid mechanical ventilation.

The peripheral nerve block (PNB) technique employed a combination of transversus abdominis plane block (TAPB) and lateral femoral cutaneous nerve block (LFCNB), using 100 mg of ropivacaine (0.5% concentration). For TAPB:

Target: The fascial plane between the internal oblique and transversus abdominis muscles; Surface landmarks: Between the iliac crest and costal margin at the midaxillary line. For LFCNB: Target: The lateral femoral cutaneous nerve (L2-L3 branches); Surface landmarks: 1–2 cm below the inguinal ligament, lateral to the sartorius muscle.

All patients received a standardized multimodal analgesia regimen, including a patient-controlled analgesia (PCA) pump with an identical formulation.

### Surgical treatment

Hemiarthroplasty is a common surgical procedure for hip fractures at our hospital and group hospitals, performed by experienced orthopedic surgeons. The procedure involves replacing only the femoral head of the hip joint while retaining the acetabulum. All patients received low-molecular-weight heparin for thromboprophylaxis (at least 10 days pre- and postoperatively, suspended 12 h before surgery) and prophylactic antibiotics pre-and postoperatively.

### Assessment and follow-up

Postoperative Delirium (POD): POD refers to acute brain dysfunction occurring within 1 week after surgery, characterized by fluctuating levels of consciousness, inattention, disorganized thinking, and cognitive impairment. Symptoms typically fluctuate over a short period and usually occur within 24–72 h postoperatively. The Confusion Assessment Method (CAM) is a commonly used tool for assessing delirium, with high validity and reliability (17). In this study, we assessed patients twice daily for the first three postoperative days using CAM to determine the occurrence of POD. CAM assessments were performed by psychiatry-trained physicians/nurses blinded to anesthesia groups. All assessors received standardized training across centers to ensure inter-rater consistency.

Delayed Neurocognitive Recovery (DNR): DNR refers to cognitive dysfunction occurring within 30 days after discharge, meeting the diagnostic criteria for mild or major neurocognitive disorder. Diagnosis relies on a series of neuropsychological tests to observe functional decline. Common assessment tools include visual reproduction, Wechsler vocabulary, digit symbol, and digit span tests. In this study, we assessed patients via telephone follow-up on the seventh postoperative day to determine the occurrence of DNR (18). The telephone-based cognitive screen was adapted from the validated Telephone Interview for Cognitive Status-Modified (TICS-M) and Inouye’s Delirium Telephone Assessment Protocol (19), with postoperative context optimizations.

Pulmonary Infection (PI): PI is inflammation of the lung tissue, usually caused by bacteria, viruses, or fungi, with common symptoms including cough, sputum production, fever, and dyspnea.

Definitive PI diagnosis required meeting ≥2 clinical criteria plus 1 imaging criterion:

Clinical criteria: Core temperature >38.3°C; Purulent sputum; Leukocyte count >12 × 10^9^/L or <4 × 10^9^/L.

Imaging criterion: New or progressive pulmonary infiltrates on chest radiograph/CT (20).

Urinary Tract Infection (UTI): UTI is an infectious disease caused by pathogen proliferation in the urinary tract, with common symptoms including urinary frequency, urgency, dysuria, and fever.

Definitive UTI diagnosis required:

≥10^3^ CFU/mL uropathogen in catheter-specimen urine culture and.

≥10 white blood cells (WBC)/high-power field (HPF) on urinalysis and.

≥1 symptom (dysuria, urgency, or suprapubic pain) (21).

Deep Vein Thrombosis (DVT): DVT is a blood clot formed in the deep veins, commonly in the lower limbs, which can cause limb swelling and pain and may lead to pulmonary embolism.

Definitive DVT diagnosis required:

Non-compressibility of venous segments on Doppler ultrasound or intraluminal filling defect on CT venography (22).

Pulmonary Thromboembolism (PTE): PTE is a blockage of the pulmonary artery or its branches by a thrombus or other material, causing pulmonary circulation impairment. Common symptoms include chest pain, dyspnea, and hemoptysis. Definitive PTE diagnosis required:

Filling defect in ≥1 subsegmental pulmonary artery on CT angiography plus D-dimer >500 μg/L (except when Wells score <2) (23).

We assessed and recorded the occurrence of the above complications, including POD, DNR, UTI, PI, DVT, and PTE, as well as mortality rates at 30 days and 6 months postoperatively. Assessments for complications (e.g., CAM for POD, telephone follow-up for DNR) were part of standard clinical protocols across all sites. CAM was routinely applied twice daily (days 1–3 postoperatively) by nursing staff, with results recorded in patient charts. DNR evaluation via telephone on postoperative day 7 was conducted by physicians as part of discharge care. PI and UTI were defined as new occurrences or worsening compared to previous conditions; DVT/PTE was defined as new occurrences or progression of thrombi.

### Statistical analysis

All statistical analyses in this study were performed using SPSS 26.0 software. For continuous variables, the Kolmogorov–Smirnov test was first used to assess whether the data followed a normal distribution. If the data were normally distributed, One-way ANOVA was used to analyze differences among the five groups; if not, the Kruskal-Wallis H test was used for non-parametric analysis. For categorical variables (count variables), the chi-square test (χ^2^ test) was used for analysis. Multiple comparisons were adjusted using the Benjamini-Hochberg false discovery rate (FDR) procedure, with odds ratios (ORs) and 95% confidence intervals (CIs) further employed to determine effect significance. Multivariable logistic regression adjusted for age, sex, BMI, operative duration, education, ASA status, and CCI to isolate anesthesia-specific effects. The significance level for all tests was set at *p* < 0.05, meaning that a *p*-value less than 0.05 was considered statistically significant.

## Results

**Figure 1:**
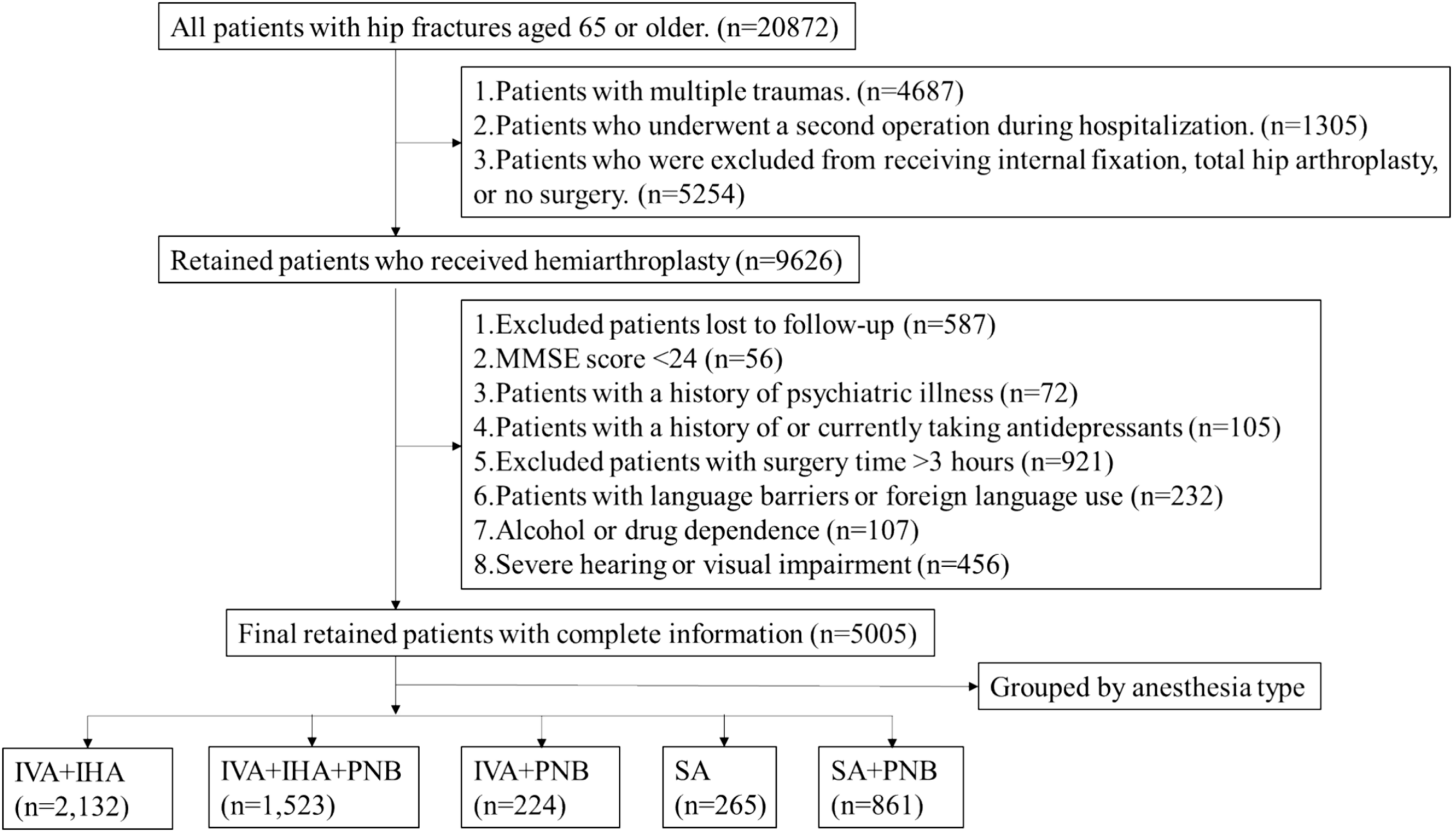
CONSORT flow. IVA, intravenous anesthesia; IHA, Inhalation anesthesia; PNB, Peripheral Nerve Block; SA, Spinal Anesthesia.

As shown in Figure 1, we initially collected 20,872 cases. Following the exclusion criteria, we excluded 9,626 patients who had multiple fractures, underwent a second surgery, or had internal fixation. This left us with patients who underwent hemiarthroplasty. Further exclusions included patients lost to follow-up, those with preoperative MMSE scores less than 24, a history of psychiatric illness, current or past use of antidepressants, surgery duration exceeding 3 h, language barriers or foreign language speakers, alcohol or drug dependence, and severe visual or hearing impairments. Ultimately, 5,005 patients met the inclusion and exclusion criteria. Among them, 2,132 patients received intravenous-inhalation general anesthesia (a), 1,523 patients received intravenous-inhalation general anesthesia combined with PNB (b), 224 patients received intravenous anesthesia combined with PNB (c), 265 patients received spinal anesthesia (d), and 861 patients received spinal anesthesia combined with PNB (e). While our overall sample size (*n* = 5,005) provided >80% power to detect clinically relevant absolute differences (≥1.8% for mortality; ≥2.5% for DVT incidence), statistical power was limited for detecting differences in rarer outcomes (e.g., PTE) within smaller subgroups (e.g., intravenous anesthesia + PNB, *n* = 224). Consequently, Type II errors cannot be excluded for these specific endpoints. Nevertheless, the consistent null findings across the majority of complications support their robustness.

### Demographic and clinical characteristics

As shown in Table 1, there were no significant differences among the five anesthesia groups [intravenous-inhalation general anesthesia (a), intravenous-inhalation general anesthesia combined with PNB (b), intravenous anesthesia combined with PNB (c), spinal anesthesia (d), and spinal anesthesia combined with PNB (e)] in terms of age, gender, body mass index (BMI), duration of surgery, years of education, American Society of Anesthesiologists Physical Status Classification (ASA) grade, and Charlson Comorbidity Index (CCI). In this study investigating the impact of different anesthetic techniques on postoperative delirium (POD) risk, we incorporated key demographic and clinical variables as covariates to control for potential confounding effects of baseline patient characteristics (Supplementary Table S1). Using multivariable logistic regression modeling, we systematically evaluated the independent associations between POD risk and the following individual factors: age, sex, BMI, operative duration, years of education, ASA classification, and Charlson Comorbidity Index (CCI). This approach aimed to quantify the effect relationships between core baseline variables and POD while controlling for their confounding effects in the analysis of anesthetic technique outcomes.

**Table 1:** Comparison of demographic data of hip fracture patients with five types of anesthesia.

Variable	a IVA + IHA	b IVA + IHA + PNB	c IVA + PNB	d SA	e SA + PNB	
	*n* = 2,132	*n* = 1,523	*n* = 224	*n* = 265	*n* = 861	
Years (Mean ± SD)	76.0 ± 5.0	75.5 ± 5.5	76.0 ± 4.5	75.0 ± 6.0	76.5 ± 5.5	*p* = 0.8589
Gender (male)	960	655	120	144	434	*p* = 0.968
BMI, kg/m^2^	24.508 ± 3.000	24.568 ± 3.047	24.488 ± 3.500	24.608 ± 3.455	24.668 ± 3.149	*p* = 0.6354
Surgical time (h)	1.75 ± 0.41	1.75 ± 0.455	1.76 ± 0.425	1.74 ± 0.475	1.75 ± 0.415	*p* = 0.1202
Years of education	7.6 ± 3.3	7.4 ± 3.5	7.4 ± 4.05	7.4 ± 3.58	7.6 ± 4.02	*p* = 0.8341
ASA grade	2.90 ± 0.135	2.91 ± 0.15975	2.89 ± 0.4158	2.92 ± 0.38295	2.88 ± 0.2124	*p* = 0.1532
CCI	2.1 ± 0.15	2.3 ± 0.194	2.2 ± 0.484	2.3 ± 0.466	2.4 ± 0.2698	*p* = 0.2564

BMI, Body Mass Index; ASA, American Society of Anesthesiologists; Patient age is in years; Gender is male/female; BMI (body mass index) is weight/height (m) squared; Operation time is in hours; Education is from primary school, recorded in years; Charlson Comorbidity Index (CCI) is per international standards; IVA, intravenous anesthesia; IHA: Inhalation anesthesia; PNB: Peripheral Nerve Block; SA: Spinal Anesthesia.

To further assess the model’s discriminative performance, we validated its predictive capability through confusion matrix analysis. Based on this, we conducted an in-depth analysis of each independent factor’s influence on POD risk. The only significant variable identified was sex (male sex showing marginally lower POD risk), though with weak effect size (OR = 0.86, 95% CI approaching 1), requiring careful clinical interpretation.

None of the other factors demonstrated statistical significance: age, BMI, operative duration, years of education, ASA classification, and CCI all failed to show significant effects.

### Incidence of postoperative delirium (POD) and delayed neurocognitive recovery (DNR)

As shown in Table 2, the incidence rates of POD were 32.83% in the intravenous-inhalation general anesthesia group (a), 32.23% in the intravenous-inhalation general anesthesia combined with PNB group (b), 28.57% in the intravenous anesthesia combined with PNB group (c), 11.69% in the spinal anesthesia group (d), and 13.05% in the spinal anesthesia combined with PNB group (e). There were no significant differences in POD incidence among the first three groups (*p* > 0.05) or between the last two groups (*p* > 0.05). However, a statistically significant difference was observed when comparing the combined first three groups with the combined last two groups (*p* < 0.05), with an overall *p*-value of 0.029. Pairwise comparisons between general anesthesia and regional anesthesia groups showed significant differences in early postoperative POD incidence. In contrast, no significant differences were observed in DNR incidence among the five groups (*p* = 0.12), indicating that anesthesia methods significantly affected the occurrence of POD but not DNR.

**Table 2:** The impact of different anesthesia methods on postoperative delirium (POD) and delayed neurocognitive recovery (DNR) in elderly hip fracture patients.

Neurocognitive outcomes	a IVA + IHA	b IVA + IHA + PNB	c IVA + PNB	d SA	e SA + PNB	
	*n* = 2,132	*n* = 1,523	*n* = 224	*n* = 265	*n* = 861	
POD	700/2132	491/1523	64/224	31/265	121/861	*p* = 0.029
	NA	Pab = 0.6749	Pac = 0.1521	Pad<0.0001	Pae < 0.0001	
	NA	NA	Pbc = 0.2241	Pbd < 0.0001	Pbe < 0.0001	
	NA	NA	NA	Pcd < 0.0001	Pce < 0.0001	
	NA	NA	NA	NA	Pde = 0.3500	
					Pabc VS de < 0.0001	
DNR	800/2132	581/1523	95/224	88/265	295/861	*P* = 0.12
	NA	Pab = 0.7009	Pac = 0.1518	Pad = 0.8635	Pae = 0.3383	
	NA	NA	Pbc = 0.2216	Pbd = 0.7180	Pbe = 0.2269	
	NA	NA	NA	Pcd = 0.2218	Pce = 0.1323	
	NA	NA	NA	NA	Pde = 0.6946	
					Pabc VS de = 0.2040	

Pabc VS de represents the comparison between general anesthesia groups (a + b + c) versus regional anesthesia groups (d + e); Pad indicates the comparison between group a and group d; IVA: intravenous anesthesia; IHA: Inhalation anesthesia; PNB: Peripheral Nerve Block; SA: Spinal Anesthesia.

The study revealed an extremely large chi-square statistic with a highly significant *p*-value (far below 0.05), indicating that the choice of anesthesia technique significantly influences the incidence of postoperative delirium (POD). After Benjamini-Hochberg false discovery rate (BH-FDR) correction (Table 3): General anesthesia (IVA + IHA) and IVA + IHA + PNB, as well as spinal anesthesia (SA) and SA + PNB, all showed *p* < 0.05, confirming a statistically significant association with POD incidence. Odds ratio (OR) analysis further demonstrated that: SA and SA + PNB had a significant protective effect against POD. IVA + IHA and IVA + IHA + PNB showed a potential trend toward increased POD risk, though with lower certainty. IVA + PNB did not show a statistically significant increase in POD risk. These findings may assist clinicians in balancing POD risk when selecting anesthesia techniques (Supplementary Table S2).

**Table 3:** Comparison of postoperative outcomes by anesthesia technique.

Anesthesia technique	Cases (n)	POD incidence	DNR incidence
IVA + IHA	2,132	700 (32.83%)	800 (37.52%)
IVA + IHA + PNB	1,523	491 (32.23%)	581 (38.15%)
IVA + PNB	224	64 (28.57%)	95 (42.41%)
SA	265	31 (11.69%)*	88 (33.21%)
SA + PNB	861	121 (13.05%)*	295 (34.26%)

POD, postoperative delirium; DNR, delayed neurocognitive recovery; IVA, intravenous anesthesia; IHA, Inhalation anesthesia; PNB, Peripheral Nerve Block; SA, Spinal Anesthesia. * Significant difference (*p* < 0.05) compared to general anesthesia groups (IVA + IHA, IVA + IHA + PNB, IVA + PNB).

Spinal Anesthesia (SA) & SA + PNB: Significant POD Risk Reduction. SA: OR = 0.3239, 67.61% lower POD risk vs. reference. 95% CI [0.2215, 0.4735] (excludes 1), statistically significant protective effect. SA + PNB: OR = 0.3634, 63.66% lower POD risk. 95% CI [0.2966, 0.4452] (excludes 1), robust protective effect. General Anesthesia (IVA + IHA & IVA + IHA + PNB): Possible but Uncertain Risk Increase. IVA + IHA: OR = 1.4976, 49.76% higher POD risk, but 95% CI [1.3229, 1.6953] includes 1, uncertain clinical significance. IVA + IHA + PNB: OR = 1.3328, 33.28% higher POD risk, but 95% CI [1.1688, 1.5198] includes 1. IVA + PNB: No Significant Risk Increase. OR = 1.3676, 36.76% higher POD risk, but 95% CI [1.0315, 1.8132] includes 1, not statistically significant. Regional anesthesia (SA/SA + PNB) should be prioritized in elderly hip fracture patients due to clear POD risk reduction. General anesthesia may slightly increase POD risk, but the evidence is less definitive; further studies are needed. IVA + PNB appears neutral, neither significantly increasing nor decreasing POD risk. This structured risk assessment can guide evidence-based anesthesia selection in clinical practice.

### Incidence of postoperative complications and mortality

As shown in Table 4, the incidence of postoperative complications and mortality across the five anesthesia groups were as follows:

**Table 4:** The impact of different anesthesia methods on postoperative pulmonary infection (PI), urinary tract infection (UI), deep vein thrombosis (DVT), pulmonary thromboembolism (PTE), 30-day and 6-month mortality in elderly hip fracture patients.

Complications	a IVA + IHA	b IVA + IHA + PNB	c IVA + PNB	d SA	e SA + PNB	
	*n* = 2,132	*n* = 1,523	*n* = 224	*n* = 265	*n* = 861	
PI	108/2132	62/1523	9/224	19/265	16/861	*P* = 0.0314
	NA	Pab = 0.8471	Pac = 0.6948	Pad = 0.0433	Pae < 0.0001	
	NA	NA	Pbc = 0.7652	Pbd = 0.0347	Pbe = 0.0002	
	NA	NA	NA	Pcd = 0.0357	Pce = 0.0231	
	NA	NA	NA	NA	Pde = 0.6767	
					Pabc VS de < 0.0001	
UTI	109/2132	89/1523	12/224	14/265	53/861	*p* = 0.0613
	NA	Pab = 0.3358	Pac = 0.8747	Pad = 0.9056	Pae = 0.2537	
	NA	NA	Pbc = 0.7709	Pbd = 0.7179	Pbe = 0.7574	
	NA	NA	NA	Pcd = 0.9710	Pce = 0.6541	
	NA	NA	NA	NA	Pde = 0.5999	
					Pabc VS de = 0.4883	
DVT	176/2132	143/1523	12/224	20/265	85/861	*p* = 0.070
	NA	Pab = 0.2311	Pac = 0.1515	Pad = 0.6918	Pae = 0.1559	
	NA	NA	Pbc = 0.1847	Pbd = 0.3366	Pbe = 0.7003	
	NA	NA	NA	Pcd = 0.2569	Pce = 0.3304	
	NA	NA	NA	NA	Pde = 0.2554	
					Pabc VS de = 0.7415	
PTE	85/2132	70/1523	10/224	15/265	40/861	*p* = 0.634
	NA	Pab = 0.3676	Pac = 0.7298	Pad = 0.1990	Pae = 0.4149	
	NA	NA	Pbc = 0.9298	Pbd = 0.4527	Pbe = 0.9558	
	NA	NA	NA	Pcd = 0.5506	Pce = 0.9082	
	NA	NA	NA	NA	Pde = 0.5032	
					Pabc VS de = 0.3634	
30-day mortality rate	107/2132	75/1523	12/224	16/265	41/861	*p* = 0.22
	NA	Pab = 0.8973	Pac = 0.8260	Pad = 0.4785	Pae = 0.7693	
	NA	NA	Pbc = 0.7812	Pbd = 0.4469	Pbe = 0.8594	
	NA	NA	NA	Pcd = 0.7475	Pce = 0.7131	
	NA	NA	NA	NA	Pde = 0.4079	
					Pabc VS de = 0.9343	
6-month mortality rate	270/2132	180/1523	30/224	38/265	105/861	*p* = 0.375
	NA	Pab = 0.4433	Pac = 0.7558	Pad = 0.4423	Pae = 0.7258	
	NA	NA	Pbc = 0.4991	Pbd = 0.2473	Pbe = 0.7857	
	NA	NA	NA	Pcd = 0.7636	Pce = 0.6289	
	NA	NA	NA	NA	Pde = 0.3597	
					Pabc VS de = 0.7709	

The numbers in the table represent the number of cases of complications, and the comparison between groups is based on the incidence rate. IVA: intravenous anesthesia; IHA: Inhalation anesthesia; PNB: Peripheral Nerve Block; SA: Spinal Anesthesia. Pabc VS de represents the comparison between general anesthesia groups (a + b + c) versus regional anesthesia groups (d + e); Pad indicates the comparison between group a and group d.

Intravenous-inhalation general anesthesia group (a): Postoperative pulmonary infection (PI): 108 patients (5.06%); Postoperative urinary tract infection (UTI): 109 patients (5.11%); Postoperative deep vein thrombosis (DVT): 176 patients (8.25%); Postoperative pulmonary thromboembolism (PTE): 85 patients (3.98%); Mortality at 30 days postoperatively: 107 patients (4.92%); Mortality at 6 months postoperatively: 270 patients (12.66%).

Intravenous-inhalation general anesthesia + PNB group (b): PI: 62 patients (4.07%); UTI: 89 patients (5.84%); DVT: 143 patients (9.39%); PTE: 70 patients (4.59%); Mortality at 30 days: 75 patients (4.92%); Mortality at 6 months: 80 patients (11.82%).

Intravenous anesthesia + PNB group (c): PI: 9 patients (4.02%); UTI: 12 patients (5.36%); DVT: 12 patients (5.36%); PTE: 10 patients (4.46%); Mortality at 30 days: 12 patients (5.36%); Mortality at 6 months: 30 patients (13.39%).

Spinal anesthesia group (d): PI: 19 patients (7.17%); UTI: 14 patients (5.28%); DVT: 20 patients (7.55%); PTE: 15 patients (5.66%); Mortality at 30 days: 16 patients (6.04%); Mortality at 6 months: 38 patients (14.34%).

Spinal anesthesia + PNB group (e): PI: 16 patients (1.86%); UTI: 53 patients (6.16%); DVT: 85 patients (9.87%); PTE: 40 patients (4.65%); Mortality at 30 days: 41 patients (4.76%); Mortality at 6 months: 105 patients (12.20%).

Significant differences were observed in the incidence of postoperative PI among the five anesthesia groups (*p* = 0.0314). General anesthesia was associated with a higher overall incidence of PI compared to regional anesthesia, with significant differences observed in pairwise comparisons between general anesthesia and regional anesthesia groups. However, no significant differences were found in the incidence of UTI, DVT, PTE, or mortality rates among the different anesthesia groups (*p* > 0.05).

The study demonstrated an exceptionally large chi-square statistic with a highly significant *p*-value (far below 0.05), indicating that anesthesia technique significantly affects PI incidence. After statistical adjustment (Table 5). General Anesthesia (IVA + IHA): *p* < 0.05, Significantly associated with higher PI risk; Spinal Anesthesia + Nerve Block (SA + PNB): *p* < 0.05, Significantly associated with lower PI risk; Other groups (IVA + IHA + PNB, IVA + PNB, SA): *p* > 0.05, No significant association with PI risk (Supplementary Table S3).

**Table 5:** Comparison of PI outcomes by anesthesia technique.

Anesthesia method	OR	95% CI	Interpretation
IVA + IHA	1.3929	[1.0590, 1.8320]	Potential PI risk increase (39.3%), but wide CI suggests low certainty
IVA + IHA + PNB	0.9297	[0.6876, 1.2571]	Neutral effect (7% lower risk), not statistically significant
IVA + PNB	0.9344	[0.4727, 1.8470]	Neutral effect (6.6% lower risk), high uncertainty (very wide CI)
SA	1.8002	[1.1048, 2.9332]	Non-significant trend toward higher PI risk (80%) (CI includes 1)
SA + PNB	0.3774	[0.2255, 0.6314]	62.3% lower PI risk with high statistical significance (CI excludes 1)

PI, pulmonary infection; IVA + IHA, general anesthesia with intravenous-inhalation agents; IVA, Intravenous anesthesia; SA, spinal anesthesia; PNB, peripheral nerve block.

Clinical Implications: SA + PNB is strongly protective, Reduces PI risk by 62% (most robust finding). IVA + IHA may increase PI risk (~39%), but confidence intervals limit certainty. Other techniques (IVA + IHA + PNB, IVA + PNB, SA alone) show no clear PI risk impact. SA alone (without PNB) paradoxically trended toward higher PI risk, although not statistically significant, these findings warrant further investigation through prospective randomized controlled trials (RCTs). For elderly hip fracture patients, SA + PNB should be prioritized to minimize pulmonary complications, while IVA + IHA may require cautious use in high-risk patients. Urinary Tract Infection (UTI), Deep Vein Thrombosis (DVT), Pulmonary Thromboembolism (PTE), 30-day Mortality, and 6-month Mortality: All chi-square statistics were non-significant (*p* > 0.05). No significant association was found between anesthesia technique and these outcomes. DVT Incidence: All anesthesia groups (IVA + IHA, IVA + IHA + PNB, IVA + PNB, SA, SA + PNB) showed *p* > 0.05. No technique significantly influenced DVT risk. OR/CI Summary (If Applicable) Note: Since no significant associations were detected, OR/CI values would likely show wide confidence intervals crossing 1, indicating no clinically meaningful effects.

## Discussion

The global aging population has led to increased incidence of hip fractures (19, 24), making anesthetic management crucial for these high-risk patients (25–27). Our multicenter study of 5,005 hemiarthroplasty patients found that general anesthesia was associated with significantly higher risks of early POD and PI compared to regional anesthesia, while no differences were observed in DNR, UTI, DVT, PTE, or mortality outcomes. Notably, intravenous anesthesia+PNB (Group c)—a regional technique with Intravenous anesthesia—showed lower POD incidence than other GA groups but higher than unsedated regional techniques (SA/SA + PNB). Despite multiplicity adjustments, residual Type I/II error risks persist in exploratory subgroup analyses (e.g., SA vs. SA + PNB). Nevertheless, core findings—increased POD/PI risk with general anesthesia (GA) versus regional techniques—remained statistically robust after rigorous correction.

The reduced POD incidence with regional anesthesia likely reflects its localized effects versus general anesthesia’s systemic neurological impact (28–31). Regional techniques avoid general anesthesia drugs that disrupt neurotransmitter balance and autonomic stability (7, 32, 33), though patient cooperation remains a consideration. Nevertheless, the REGAIN trial (34) found no statistically significant difference in delirium incidence between spinal and general anesthesia. This discrepancy may arise from: (i) REGAIN’s relatively younger cohort; (ii) Protocolized delirium prevention in REGAIN’s GA group (e.g., EEG-guided anesthesia); (iii) Our study primarily examined early perioperative cognitive dysfunction, specifically postoperative delirium (POD) within 72 h and delayed neurocognitive recovery (DNR) on postoperative day 7.

The equivalent DNR rates across groups suggest anesthesia type primarily affects early postoperative cognition. Mid-term recovery appears more influenced by patient factors like comorbidities and rehabilitation. For PI, general anesthesia’s respiratory depression, pro-longed drug metabolism, and airway instrumentation increase risk (10, 35–38), while regional methods preserve respiratory function (39, 40).

The lack of difference in other complications may reflect modern perioperative protocols effectively managing these risks (19, 41). While mortality showed no variation, longer-term studies are needed.

## Strengths and limitations of the study

The strengths of this study include its large sample size and multicenter data source, which provide high representativeness and reliability. Additionally, the follow-up of multiple complications and different time points offers a comprehensive assessment of the actual risks of different anesthesia methods on perioperative complications in elderly patients. However, there are also some limitations. This study is a retrospective observational study with potential selection bias and the inability to completely exclude the influence of confounding factors. We acknowledge unmeasured confounders including frailty (e.g., Clinical Frailty Scale) and granular cognitive profiles. While ASA/CCI captured major comorbidities, anesthesia selection bias in this non-randomized cohort reflecting clinical judgment remains a limitation. Additionally, while our telephone battery demonstrated good validity against MoCA and greater feasibility, it remains less comprehensive than in-person neuropsychological testing.

While anesthesia techniques were controlled, quantitative analysis of dose-dependent drug effects (e.g., propofol vs. sevoflurane neurotoxicity, ropivacaine plasma kinetics) was precluded. Retrospective quantification was infeasible due to: (i) protocolized dosing limiting pharmacological variability, and (ii) absence of drug-level monitoring (e.g., plasma concentrations, depth-of-anesthesia metrics). Future pharmacodynamic studies should correlate anesthetic exposure (AUC, Cmax) with PND/PI outcomes. Although early delayed neurocognitive recovery (DNR) was assessed via validated telephone-adapted tools (TICS-M/Inouye protocol) (19), deficits emerging post-discharge (days 8–30) were unevaluated, potentially underestimating true incidence.

## Conclusion

In summary, this study suggests that for elderly patients with hip fractures, regional anesthesia may be more beneficial than general anesthesia in reducing the risk of early postoperative POD and PI. However, no significant differences were observed in DNR and other complications or mortality rates. These findings have important implications for clinical practice: when devising anesthesia plans, especially for high-risk elderly patients, it is necessary to weigh the impact of anesthesia methods on perioperative neurocognitive function and complications to develop individualized perioperative management strategies. Future research could further investigate the effects of different anesthesia drugs, doses, methods, and other perioperative management measures on perioperative neurocognitive disorders and prognosis in elderly patients with hip fractures through multicenter, prospective, randomized controlled clinical trials, in order to provide more precise and safer anesthesia management solutions for clinical practice.

## Data Availability

The original contributions presented in the study are included in the article/Supplementary material, further inquiries can be directed to the corresponding author.
