# Supplementary material for: A multicenter retrospective study on anesthesia methods and their impact on neurocognitive outcomes and other complications in elderly patients undergoing hemiarthroplasty
Source: Front Med (Lausanne). 2025 Aug 11;12:1599989. doi: 10.3389/fmed.2025.1599989 (PMC12375961; doi:10.3389/fmed.2025.1599989)
Supplement: Supplementary file 1 [file Table_1.docx]

Supplementary Table 1. Multivariable Logistic Regression Analysis of Factors Associated with Postoperative Delirium (POD)

| Variable | Coefficient (coef) | Odds Ratio (OR) | 95% CI Lower | 95% CI Upper | Statistical Significance |
| --- | --- | --- | --- | --- | --- |
| Constant (const) | -0.9240 | 0.397 | -2.299 | 0.451 | Not significant |
| Age (years) | 0.0052 | 1.005 | -0.009 | 0.019 | Not significant |
| Sex (Male=1) | -0.1494 | 0.861 | -0.298 | -0.001 | Marginal significance |
| BMI | 0.0068 | 1.007 | -0.017 | 0.031 | Not significant |
| Operative duration (h) | -0.1273 | 0.880 | -0.360 | 0.105 | Not significant |
| Education (years) | -0.0232 | 0.977 | -0.051 | 0.005 | Not significant |
| ASA classification | 0.0100 | 1.010 | -0.081 | 0.101 | Not significant |
| Charlson Comorbidity Index (CCI) | -0.0462 | 0.955 | -0.194 | 0.102 | Not significant |

Notes: BMI: Body Mass Index; ASA: American Society of Anesthesiologists; PNB: Peripheral Nerve Block; Patient age is in years; Gender is male/female; BMI is weight/height (m) squared; Operation time is in hours; Education is from primary school, recorded in years; Charlson score is per international standards. CI = confidence interval；Reference categories: Female for sex variable；Marginal significance defined as p-value between 0.05-0.10；All continuous variables were analyzed per unit increase；OR >1 indicates increased risk, OR <1 indicates decreased risk
