# Supplementary material for: A multicenter retrospective study on anesthesia methods and their impact on neurocognitive outcomes and other complications in elderly patients undergoing hemiarthroplasty
Source: Front Med (Lausanne). 2025 Aug 11;12:1599989. doi: 10.3389/fmed.2025.1599989 (PMC12375961; doi:10.3389/fmed.2025.1599989)
Supplement: Supplementary file 2 [file Table_2.docx]

| Outcome | Test | **χ²** Statistic | *P*-value | BH-FDR Adjusted p-values* | Significant (α=0.05) |
| --- | --- | --- | --- | --- | --- |
| POD | Chi-square | 155.91 | 1.10 × 10⁻³² | [4.77 × 10⁻¹⁰, 2.54 × 10⁻⁵, 9.36 × 10⁻¹, 2.61 × 10⁻⁹, 4.94 × 10⁻²³] | [T, T, T, T, T] |
| DNR | Chi-square | 8.27 | 0.082 | [0.652, 0.433, 0.275, 0.324, 0.275] | [F, F, F, F, F] |

Supplementary Table 2. Statistical Analysis of Postoperative Outcomes by Anesthesia Type.

POD =postoperative delirium; DNR = delayed neurocognitive recovery; BH-FDR = Benjamini-Hochberg false discovery rate correction applied to multiple comparisons. T = True (significant), F = False (not significant).
