# Supplementary material for: A multicenter retrospective study on anesthesia methods and their impact on neurocognitive outcomes and other complications in elderly patients undergoing hemiarthroplasty
Source: Front Med (Lausanne). 2025 Aug 11;12:1599989. doi: 10.3389/fmed.2025.1599989 (PMC12375961; doi:10.3389/fmed.2025.1599989)
Supplement: Supplementary file 3 [file Table_3.docx]

| Outcome Measure | χ² Statistic | Original *P*-value | BH-FDR Adjusted *P*-values* | Significant (α=0.05) |
| --- | --- | --- | --- | --- |
| Pulmonary Infection (PI) | 21.16 | 2.94 × 10⁻⁴ | [0.042, 0.864, 0.979, 0.042, 8.47 × 10⁻⁴] | [T, F, F, T, T] |
| Urinary Tract Infection (UTI) | 1.69 | 0.793 | [0.953, 0.953, 1.000, 1.000, 0.953] | [F, F, F, F, F] |
| Deep Vein Thrombosis (DVT) | 6.52 | 0.164 | [0.437, 0.437, 0.437, 0.563, 0.437] | [F, F, F, F, F] |
| Pulmonary Thromboembolism (PTE) | 2.13 | 0.711 | [0.950, 0.953, 1.000, 0.950, 0.953] | [F, F, F, F, F] |
| 30-day Mortality | 0.78 | 0.941 | [1.000, 1.000, 1.000, 1.000, 1.000] | [F, F, F, F, F] |

Supplementary Table 3. Statistical Analysis of Postoperative Complications and Mortality.

BH-FDR = Benjamini-Hochberg false discovery rate correction. T = True (significant at α=0.05), F = False (not significant).
